# Effect of Fish-Derived Lipids on Inflammation Status and Health-Related Quality of Life in Women with Endometriosis

**DOI:** 10.3390/nu18132184

**Published:** 2026-07-05

**Authors:** Angelika Bogusz, Kacper Szewczyk, Dariusz Włodarek, Magdalena Górnicka

**Affiliations:** 1 Department of Human Nutrition, Institute of Human Nutrition Sciences, Warsaw University of Life Sciences (SGGW-WULS), 166 Nowoursynowska St., 02-787 Warsaw, Poland; kacper_szewczyk@sggw.edu.pl; 2 Department of Dietetics, Institute of Human Nutrition Sciences, Warsaw University of Life Sciences (SGGW-WULS), 166 Nowoursynowska St., 02-787 Warsaw, Poland; dariusz_wlodarek@sggw.edu.pl

**Keywords:** endometriosis, fish-derived lipids, *n*-3, alkylglycerol, squalene, inflammation, pain, endometriosis quality of life

## Abstract

**Background**: Endometriosis is a chronic, estrogen-dependent inflammatory disorder associated with pain and immune dysregulation. Omega-3 (*n*-3) fatty acids and fish-derived bioactive lipids may modulate inflammation and metabolism. This study investigated whether adding fish-derived lipids (FDLs) to a Healthy Eating Plate diet improves inflammatory markers, gut inflammation, pain, and quality of life in women with endometriosis. **Methods**: In this 12-week randomized controlled trial, 46 women with confirmed endometriosis were assigned to either a Healthy Eating Plate diet alone (control group, CG) or the same diet plus FDLs (intervention group, IG). Primary outcomes included serum cytokine concentrations (interleukin [IL]-1β, IL-6, IL-8, IL-10, and tumor necrosis factor-alpha [TNF-α]) and fecal calprotectin (CAL). Secondary outcomes included pain intensity measured using the visual analog scale (VAS) and health-related quality of life assessed with the Endometriosis Health Profile-30 (EHP-30). **Results**: IL-10 levels (pg/mL) increased in the IG (32.5 ± 51.5 to 265 ± 508; *p* = 0.024) with a significant adjusted effect (ANCOVA: 5.07 [95% CI: 1.32–19.48], *p* = 0.019). Other cytokines showed heterogeneous responses, and CAL levels remained unchanged. EHP-30 Pain scores improved within both groups (CG *p* = 0.016; IG *p* = 0.002) without significant between-group change or adjusted effects (all *p* > 0.05). VAS scores decreased within the IG (5.0 ± 2.1 to 4.3 ± 2.1; *p* = 0.011), although there were no between-group differences in change and ANCOVA (all *p* > 0.05). However, ≥1-point responder rates were higher in the IG vs. CG (73.9% vs. 34.8%): adjusted RR = 2.11 (95% CI: 1.16; 3.89; *p* = 0.014). **Conclusions**: Overall, FDL supplementation was associated with a selected regulatory immune signal, reflected mainly by the IL-10 response, while most inflammatory, pain-related, and quality-of-life outcomes did not show consistent significant between-group effects. The observed greater likelihood of pain reduction in the FDL-supplemented group of women with endometriosis, along with the IL-10 response, should be interpreted with caution and confirmed in larger studies with longer follow-up/intervention time, taking into account the potential mechanisms of biological response.

## 1. Introduction

Endometriosis is a common, estrogen-dependent chronic gynecologic disease defined by the presence of endometrial-like tissue outside the uterine cavity [1]. Beyond the traditional pelvic framework, endometriosis is increasingly conceptualized as a chronic inflammatory and immune-mediated condition in which chronic inflammation and immune dysregulation contribute to lesion establishment, persistence, and symptom expression [2,3]. The symptom spectrum is dominated by chronic pelvic pain, dysmenorrhea, dyspareunia, and dyschezia, contributing to substantial impairment in functioning and health-related quality of life (QoL) [2]. The etiology is multifactorial and complex and remains largely unknown [4,5].

From an immunological perspective, endometriosis involves dysregulated inflammatory signaling across both innate and adaptive immune pathways [2,6]. Cytokines and chemokines coordinate leukocyte recruitment, immune cell activation, angiogenesis, neuroinflammation, and tissue remodeling within the peritoneal environment and lesions [2,3,7,8]. Pro-inflammatory cytokines such as IL-1β, IL-6, IL-8 and TNF-α may amplify local inflammatory signaling, promote lesion survival and vascularization, and contribute to nociceptive sensitization and pain-related mechanisms [2,3,6,8,9,10]. However, the inflammatory phenotype of endometriosis is not limited to pro-inflammatory activation alone. Anti-inflammatory and immunoregulatory cytokines, particularly IL-10, may constrain excessive immune activation and support immune homeostasis. However, elevated IL-10 has also been described as part of a more immunosuppressive microenvironment that may favor the persistence of ectopic endometrial-like tissue [6,11,12]. Therefore, the assessment of both pro-inflammatory and anti-inflammatory cytokine responses may provide a more comprehensive view of the immune-inflammatory status in women with endometriosis [11,12].

Gastrointestinal symptoms are common in endometriosis, and emerging work highlights potential gut–immune interactions that may influence systemic inflammatory tone. Fecal calprotectin (CAL) is a validated, non-invasive marker of neutrophil-driven intestinal mucosal inflammation in inflammatory bowel disease; however, it is not a marker of endometriosis activity per se [13,14,15,16]. In this study, CAL was included as an exploratory, non-specific indicator of gut inflammatory activity in the context of prevalent gastrointestinal complaints and possible bidirectional gut–immune signaling.

Current clinical management relies primarily on pharmacologic and surgical interventions, with hormonal therapies remaining central for symptom control [17]. Alongside standard care, modifiable lifestyle factors, particularly diet, are increasingly discussed as adjunct strategies that may influence endocrine and inflammatory pathways relevant to endometriosis [18,19]. Dietary fat quality, specifically the balance between saturated (SFA), monounsaturated (MUFA), and polyunsaturated fatty acids (PUFA), may differentially affect inflammatory signaling and metabolic regulation [20]. In experimental and metabolic contexts, SFAs can activate toll-like receptor 4 (TLR4) and downstream nuclear factor kappa B (NF-κB) signaling, promoting pro-inflammatory cytokine production [21]. However, MUFAs have been linked to peroxisome proliferator-activated receptor (PPAR) activation, and a more favorable adipokine/cytokine profile, including higher adiponectin [22]. PUFAs serve as substrates for eicosanoid synthesis, and lipid mediators (including prostaglandins) may exert distinct inflammatory effects depending on the precursor fatty acid [23,24].

FDLs provide bioactive lipid classes, primarily marine *n*-3 PUFA eicosapentaenoic acid (EPA) and docosahexaenoic acid (DHA), and, in some preparations, additional constituents such as alkylglycerols and squalene. These lipid classes have been reported to modulate immune and inflammatory pathways. Importantly, FDL formulations may exert biological effects related to the broader lipid matrix rather than to isolated *n*-3 fatty acids alone [25]. However, evidence in endometriosis populations remains limited. EPA and DHA are widely recognized for immunomodulatory actions and may influence inflammatory mediator production and pain-related pathways [26,27,28]. Squalene and alkylglycerols have also been reported to affect inflammatory responses in experimental contexts, although clinical evidence in endometriosis remains limited [29,30]. Observational studies suggest that higher *n*-3 PUFA exposure may be associated with lower endometriosis risk, whereas interventional findings for symptom improvement are heterogeneous [31,32]. Accordingly, the molecular effects of *n*-3 PUFA-rich interventions in women with endometriosis remain insufficiently characterized, supporting the need for controlled trials incorporating objective exposure measures and mechanistically informative endpoints.

The aim of this study was to compare the effects of FDL supplementation added to a Healthy Eating Plate-based diet with diet alone on inflammatory status and quality of life in women with endometriosis. The main research question was whether adding FDLs as a source of biologically active compounds, such as PUFAs, alkylglycerols, and squalene, to the diet would be associated with different changes in inflammation and immune system activation biomarkers, fecal calprotectin (CAL), pain intensity, and health-related quality of life compared with diet alone. Circulating cytokines were assessed as immune-related outcomes, while fecal CAL was included as an exploratory marker of gut inflammatory activity. Pain intensity and health-related quality of life were evaluated using the visual analog scale (VAS) and the Endometriosis Health Profile-30 (EHP-30), respectively. Given the exploratory nature of the study, the analyses were intended to generate hypotheses regarding possible immunomodulatory responses rather than to establish clinical efficacy.

## 2. Materials and Methods

This study was a prospective, two-arm, parallel, controlled dietary intervention conducted over a 12-week period to evaluate the effect of a biological fish oil composition combined with a standardized diet versus diet alone. The research was carried out at the Institute of Human Nutrition Science, Warsaw University of Life Sciences (SGGW), between April 2024 and May 2025.

The study protocol was approved by the Rector’s Committee on Human Research Ethics of Warsaw University of Life Sciences (number 5/RKE/2024U). It was conducted in accordance with all procedures adhering to the ethical standards for human research. All participants provided written informed consent prior to enrollment. Clinical trial registration was not required prospectively under the applicable local requirements for this non-drug dietary supplementation study.

Participants were recruited online using a physician-administered screening questionnaire. Eligible women were invited to an in-person meeting at the Institute, where study procedures were explained and informed consent was obtained.

### 2.1. Participants

**Figure 1:**
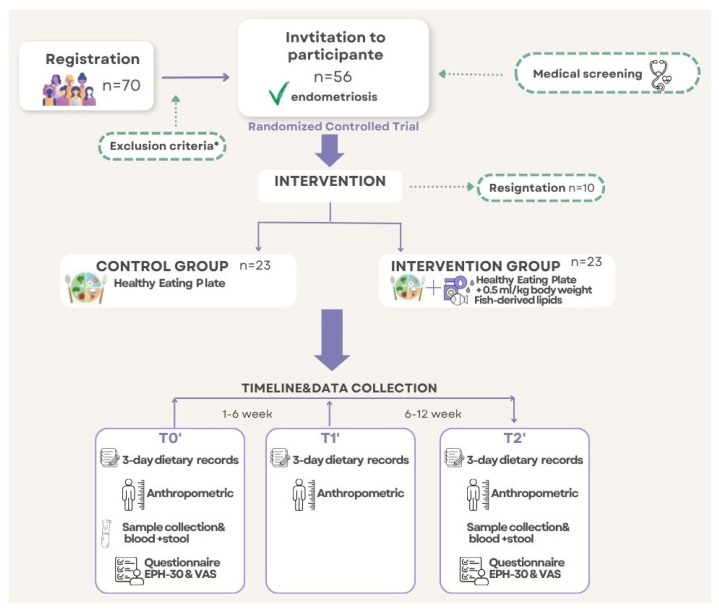
Study flowchart: study design, allocation, and sample collection schedule (created with Canva, Pty Ltd., Sydney, Australia; https://www.canva.com/). * Exclusion criteria: (1) pregnancy or breastfeeding; (2) menopause or postmenopausal status; (3) liver, kidney, or cardiac diseases; (4) acute inflammatory disorders of the intestines, pancreas, liver, or gallbladder; (5) fish allergy; (6) epilepsy; (7) implanted medical devices (e.g., pacemaker, defibrillator, stent, metallic sutures, or prosthetic implants); (8) history of radiotherapy or chemotherapy; (9) medical conditions requiring a specialized diet; and (10) intake of dietary supplements containing *n*-3, *n*-6, or *n*-9 fatty acids. Solid purple arrows indicate the main study flow and allocation process. Green dashed arrows and boxes indicate screening-related steps, exclusion criteria, and resignation. T0, baseline; T1, week 6; T2, week 12.

A total of 56 women aged 22–48 years with a confirmed diagnosis of endometriosis were enrolled (Figure 1). Forty-six participants completed the full 12-week intervention. Inclusion criteria were (1) confirmed endometriosis diagnosis, and (2) female sex, age 18–48 years. Exclusion criteria comprised: (1) pregnancy or breastfeeding; (2) menopause or postmenopausal status; (3) liver, kidney, or cardiac diseases; (4) acute inflammatory disorders of the intestines, pancreas, liver, or gallbladder; (5) fish allergy; (6) epilepsy; (7) implanted medical devices (e.g., pacemaker, defibrillator, stent, metallic sutures, or prosthetic implants); (8) history of radiotherapy or chemotherapy; (9) medical conditions requiring a specialized diet; and (10) intake of dietary supplements containing *n*-3, *n*-6, or *n*-9 fatty acids. Data such as age, place of living, education, physical activity level, medical history, dietary habits, and supplement use were collected. Women were eligible for inclusion if they had a previously confirmed diagnosis of endometriosis documented in their medical records. Diagnostic confirmation was verified by a study physician during the screening process before enrollment. Depending on the participant’s previous diagnostic pathway, the diagnosis was based on specialist gynecological assessment supported by clinical evaluation, imaging findings, and/or surgical documentation. Women who met the inclusion criteria and provided written informed consent were enrolled in the study and randomly assigned to the control group (CG; *n* = 23) or the intervention group (IG; *n* = 23). Randomization was stratified by BMI value (<25 vs. ≥25 kg/m^2^).

Before the baseline, each participant underwent a medical screening visit with a study physician. This included a physical examination and a review of the participant’s medical history to exclude any contraindications to participation and confirm their general health status.

### 2.2. Study Intervention

Women in the CG followed the Polish Healthy Eating Plate diet, while the IG followed the same diet plus FDL supplementation at a dose of 0.5 mL/kg body weight/day for 12 weeks. Both groups received individualized dietary counseling based on the Polish Healthy Eating Plate model, which recommends that meals consist of half vegetables and fruit, one quarter whole-grain products, and one quarter protein sources, with the addition of plant-based oils, and the limitation of trans fats, highly processed foods, and sugar-sweetened beverages. Water, tea, and coffee were permitted as the main beverages, and regular physical activity was encouraged. Individualized 7-day meal plans were provided at baseline and after six weeks by a clinical dietitian. All participants in the IG received the same commercially manufactured FDL product throughout the 12-week intervention. The formulation consisted of a complex mixture of approximately 500 bioactive lipids, including defined amounts of *n*-3, *n*-9, and *n*-11 fatty acids, alkylglycerols, squalene, vitamin A, and vitamin D3. The major quantified components were DHA (21.7 g/100 g of product), EPA (11.4 g/100 g), oleic acid (13.0 g/100 g), palmitic acid (6.2 g/100 g), gondoic acid (3.8 g/100 g), cetoleic acid (3.5 g/100 g), docosapentaenoic acid (2.8 g/100 g), palmitoleic acid (2.3 g/100 g), squalene (8.8 g/100 g), and alkylglycerols (8.1 g/100 g). The full compositional profile is provided in Supplementary Table S1. The daily FDL dose was 0.5 mL/kg body weight and was administered in two divided doses per day.

Adherence to dietary recommendations and supplementation was monitored using 3-day food records collected at T0 and T2. To reduce dropout, a supportive study environment was maintained, laboratory visits were scheduled flexibly (±3 days), and email reminders were sent. Participants received complete study results, and study staff contacted them as agreed to monitor adherence and address any difficulties. Additional support was provided through counseling by a clinical dietitian and educational webinars conducted during the intervention to enhance adherence. To document the intake of the studied products, participants kept a diary in which they recorded all medications, herbal products, dietary supplements, and foods for special medical purposes used during the study period. In addition, during the follow-up visits, they were questioned about their self-assessment of compliance with the recommendations to ingest 0.5 mL/kg body weight of FDLs.

### 2.3. Dietary Assessment

Dietary intake was assessed using 3-day weighed food records collected prospectively during the week preceding the baseline visit and the week preceding the final visit. Each record included two weekdays and one weekend day. Participants received both verbal and written instructions on how to record all foods and beverages consumed, including detailed information on portion sizes, preparation methods, and brand names where applicable. At each study visit, the research team reviewed the records for completeness and clarified any ambiguities. To enhance the accuracy of portion size estimation, dietary records were verified during follow-up visits through structured interviews conducted by a clinical dietitian using the Album of Photographs of Food Products and Dishes (National Institute of Public Health—National Research Institute, Poland). Data from the dietary records were entered into the ALIANT Pro Dietetics software (Gdansk, Poland, https://aliant.com.pl/) by a dietitian trained in dietary assessment to estimate energy and nutrient intake. Mean daily energy and nutrient intake was calculated for each participant at each assessment point. Dietary adequacy was evaluated by comparing individual intakes with the Polish Dietary Reference Values (DRVs) [33]. Macronutrient intake was expressed as a percentage of total energy intake and compared with reference intake (RI) values: protein 10–20%, carbohydrates 45–65%, and total fat 30–40%. Fat quality was assessed based on fatty acid composition: SFAs comprised <10% of total energy intake [33], MUFAs 10–20% [34], and PUFAs 6–11%, in line with current dietary guidelines [35]. Long-chain *n*-3 fatty acid (EPA + DHA) adequacy was assessed by comparing daily intake with AI level (250 mg/day for adults) [33].

### 2.4. Anthropometric Measurements

Body height and body weight were measured to calculate body mass index (BMI), expressed as body weight in kilograms divided by height in meters squared (kg/m^2^). Body height was measured using a stadiometer (Seca, Hamburg, Germany), and body weight was assessed with a digital scale (SECA 515 mBCA, Hamburg, Germany). Waist and hip circumferences were measured using a flexible, non-elastic measuring tape (Seca 203, Hamburg, Germany). Waist circumference was measured at the midpoint between the lower margin of the last palpable rib and the top of the iliac crest, while hip circumference was measured at the level of the greatest posterior protuberance of the buttocks, with the tape positioned horizontally around the body. These measurements were used to calculate the waist-to-hip ratio (WHR) and the waist-to-height ratio (WHtR): WHR = waist circumference (cm)/hip circumference (cm); WHtR = waist circumference (cm)/body height (cm). All anthropometric measurements were performed in the Anthropometrics Laboratory by trained researchers in accordance with standardized procedures and protocols described in the National Health and Nutrition Examination Survey (NHANES) guidelines [36]. Measurements were conducted with participants wearing light clothing and without shoes. Each anthropometric measurement was taken in duplicate. If the difference between the two measurements exceeded 10%, a third measurement was performed, and the mean value of the two closest measurements was used in the analysis.

### 2.5. Body Composition

The analysis of body composition was performed using the bioelectrical impedance method (BIA) according to manufacturer protocols by a qualified person. The following measurement procedures were maintained for each participant: refraining from vigorous physical activity for at least 12 h before the test; no caffeine and alcohol consumption for 24 h before the test; avoiding certain medications such as diuretics, laxatives and electrolyte replacement drugs; fasting for 4 h after a meal; emptying the bladder 30 min before the test; and removing any metal jewelry. Body Composition Analyzer InBody 770 (Inbody Co., Ltd., Seoul, Republic of Korea), with Lookin’Body 120, version 3.0.0.11 software, was used to estimate body fat content (%).

### 2.6. Biochemical Analysis

#### 2.6.1. Sample Collection

Prior to the collection of baseline blood and fecal samples, each participant underwent a medical screening visit conducted by a study physician. The screening encompassed a comprehensive review of the subject’s medical history, with the objective of excluding any contraindications to participation, as well as confirming their eligibility and overall health status. Participants who satisfied all the inclusion criteria and passed the medical screening proceeded to the baseline biochemical assessments (T0). Blood samples were collected on two occasions by qualified personnel in a certified medical diagnostic laboratory: before the intervention (T0, baseline) and after completion of the intervention (T2, final visit). Participants were required to attend the laboratory in the morning following an overnight fast. On the same examination days, morning fecal samples were also collected in accordance with standardized procedures. All collected biological samples from the baseline and final visits were analyzed by the Medical Diagnostic Laboratory in Łódź, Poland, in accordance with the laboratory’s standard operating procedures.

#### 2.6.2. Biomarkers of Inflammatory Status

Peripheral blood lymphocytes were isolated by density-gradient centrifugation using Lymphoprep (1.077 g/cm^3^), washed with PBS and suspended in RPMI-1640 supplemented with 2 mM L-glutamine, 10% FCS and antibiotics (1 × 10^6^ cells/mL). Cells (1 × 10^5^/well) were cultured for 24 h (37 °C, 5% CO_2_) under two conditions, unstimulated (control) and stimulated with PHA (5 μg/mL), each in triplicate. Cytokine pro-inflammatory (IL-1β, IL-6, IL-8, TNF-α) and anti-inflammatory (IL-10) culture supernatants were quantified using the BD CBA Human Inflammatory Cytokine Kit and a FACSCanto cytometer, and analyzed with FCAP Array v 3.0. Results are expressed as pg/mL or ng/mL.

#### 2.6.3. Calprotectin

Fecal CAL was measured using the LIAISON^®^ Calprotectin assay (DiaSorin; REF 318960), a quantitative two-site chemiluminescent immunoassay (CLIA) performed on LIAISON^®^ analyzers, and reported as µg/g stool. Stool was collected in a clean container without preservatives and stored at 2–8 °C until processing (stability: ≤6 h at room temperature or ≤72 h at 2–8 °C); if not analyzed within 72 h, samples were frozen at ≤−20 °C (up to 16 weeks). CAL was extracted with LIAISON^®^ Q.S.E.T. Buffer: 50–100 mg of stool was mixed with 1× buffer (49 *w*/*v* equivalents; ~2.5–4.9 mL depending on stool mass) and homogenized for 30 min; 1.0 mL of homogenate was centrifuged (5 min, 3000× *g*), and 100 µL supernatant was diluted with 850 µL of 1× buffer prior to analysis. The assay measuring range was 5–800 µg/g (values < 5 µg/g reported as <5 µg/g); samples above the range were re-tested using 1:10 on-instrument dilution with the manufacturer’s diluent. Manufacturer decision points were <50 µg/g (normal), 50–120 µg/g (borderline), and >120 µg/g (elevated).

### 2.7. Endometriosis Health Profile-30 (EHP-30)

The health-related quality of life of the women was assessed using the EHP-30, a disease-specific questionnaire that has been validated for the purpose of measuring the impact of endometriosis on various aspects of physical, emotional, and social well-being. Women could choose from 1 of 5 response categories: never (0); rarely (1), sometimes (2), often (3) and always (4). The study encompasses five subscales, which are designed to assess various aspects of the experience, including “pain”, “Control and powerlessness”, “Social support”, “Emotional well-being” and “Self-image”. The scale ranges from 0 to 100, with higher values denoting superior performance [37]. The EHP-30 questionnaire was administered online at baseline, T0, and T2. A clinically meaningful response was defined as a reduction in VAS pain intensity from T0 to T2 meeting prespecified thresholds (≥1 and ≥2 points).

### 2.8. Pain Intensity Assessment

The intensity of pain was measured using the VAS, a tool that has been validated and is widely used to assess pain. This scale enables subjects to evaluate their perceived pain on a continuous scale ranging from 0 (no pain) to 10 (extreme pain). The VAS questionnaire was administered online at T0 and T2.

### 2.9. Statistical Analysis

Statistical analyses were performed using Statistica version 20 (TIBCO Software Inc., Palo Alto, CA, USA). All tests were two-sided and statistical significance was set at *p* < 0.05 unless stated otherwise. Continuous variables are presented as mean ± SD for approximately symmetric distributions and as the median [IQR] for skewed distributions; categorical variables are presented as *n* (%). The primary analytic set comprised participants with complete T0 and T2 data (complete-case/per-protocol analysis). For fecal CAL, due to incomplete laboratory measurements, analyses were conducted using available cases without imputation; no missing-data imputation was applied. Within-group comparisons were performed using a paired *t*-test or Wilcoxon signed-rank test depending on the normality of differences (Shapiro–Wilk test). Between-group differences in change scores (Δ = T2 − T0) were assessed using an independent *t*-test/Welch test or Mann–Whitney *U* test. These analyses were not used to determine covariate inclusion and were considered descriptive. Between-group differences in outcomes at T2 were evaluated using analysis of covariance (ANCOVA) with treatment group as a fixed factor and the baseline value of the respective outcome (T0) as a covariate (Outcome_T2 − Group + Outcome_T0). Adjusted group effects are reported as estimates with 95% confidence intervals (CIs) and *p*-values. Within-group tests (paired *t* tests/Wilcoxon) and unadjusted comparisons of change scores were used, if reported, as descriptive summaries only and were not used for primary inference about treatment effects. For cytokines and CAL with pronounced right skewness, analyses were performed on the log-transformed scale. To enable log-transformation and retain all observations, a biomarker-specific offset was added prior to transformation (ln[value + offset]). Effects from log-scale models are presented as ratios (fold-changes) with a 95% CI. Biomarker-specific offsets were defined as statistical continuity constants, rather than clinical thresholds, and were selected as one-half of the smallest positive observed value for each biomarker across T0 and T2 measurements. The same marker-specific offsets were applied consistently across ANCOVA, regression and correlation analyses. A responder was defined as achieving a clinically meaningful reduction in pain intensity from T0 to T2 using prespecified thresholds (e.g., ≥1 and ≥2 points on the VAS). Between-group differences in responder rates were assessed using generalized linear models to estimate risk ratios (RRs) with 95% confidence intervals, with optional adjustment for baseline pain. Associations between changes in Δdietary exposures and changes in clinical and biomarker outcomes were considered exploratory. In addition to regression modeling, Spearman rank correlations (ρ) were computed to evaluate monotonic associations between Δ dietary variables and Δcytokines/CAL. Correlations were calculated for the full sample (*n* = 46). For cytokines and CAL, Δvalues used in correlation analyses were derived on the log-transformed scale as Δ = ln(T2 + offset) − ln(T0 + offset), using the same marker-specific offsets as above. Linear regression models were fitted with Δ outcome as the dependent variable and ΔExposureas the predictor, including group as a covariate and testing effect modification using a Group × ΔExposure interaction term; where the interaction was nominally significant or borderline, group-specific slopes are reported.

## 3. Results

### 3.1. Participants’ Characteristics

Table 1 shows the baseline characteristics of the study participants. The mean age was 34 years and did not differ significantly between groups. The majority of women had been diagnosed more than 12 months prior in both groups (62% in the CG and 61% in the IG). Most participants had higher education (91% in both groups) and lived in large cities (78% in the CG and 87% in the IG). No statistically significant differences were observed between groups for any baseline characteristics.

### 3.2. Intervention and Changes in Diet

#### 3.2.1. Energy and Macronutrients

No significant within-group changes were observed in the CG from T0 to T2 for protein, carbohydrates, total fat (% energy) and total energy value (all *p* > 0.05) (Table 2). In relation to RI, total fat in the CG remained within the RI, whereas carbohydrate intake was below the RI range at both time points. Mean value as a % of energy from protein was close to the upper RI bound and remained stable. In the IG, carbohydrate intake (% energy) decreased and total fat intake (% energy) increased (both *p* < 0.001); protein (% energy) remained unchanged, while total energy value increased (*p* = 0.013). Between-group differences in change (Δ) were significant for carbohydrates and total fat (both *p* < 0.001), but not for protein or energy intake (both *p* > 0.05). ANCOVA confirmed significant adjusted intervention effects for carbohydrates (adj. effect (IG-CG): −7.21; 95% CI: −9.71 to −4.71; *p* < 0.001) and total fat (adj. effect (IG-CG): 7.86; 95% CI: 5.43 to 10.29; *p* < 0.001), with no significant adjusted effects for protein and total energy intake (all *p* > 0.05) (Table 2).

#### 3.2.2. Adequacy of Fat and Fatty Acid Intake

Within-group analyses showed no significant changes in SFA intake in either the CG or the IG (all *p* > 0.05) (Table 3). In the CG and IG, MUFA intake increased significantly from T0 to T2 (CG: *p* = 0.011 IG: *p* < 0.001). PUFA intake increased significantly in the IG (*p* < 0.001), whereas no significant within-group change was observed in the CG (*p* > 0.05). No significant within-group changes were observed for *n*-6 in either group (all *p* > 0.05). Omega-3 intake increased within both groups (CG: *p* = 0.006; IG: *p* < 0.001), with a markedly larger increase in the IG. EPA + DHA intake also increased within both groups (CG: *p* = 0.021; IG: *p* < 0.001). Between-group comparisons of change (Δ) were significant for PUFA, *n*-3 and EPA + DHA (all *p* < 0.001), whereas no significant between-group differences were observed for SFA, MUFA and *n*-6 (both *p* > 0.05). ANCOVA confirmed significant adjusted intervention effects for SFA (*p =* 0.034) PUFA, *n*-3 and EPA + DHA (all *p* < 0.001), while no significant adjusted effects were observed for MUFA and *n*-6 (all *p* > 0.05).

In IG, significant increases in the intakes of alkylglycerols, squalene, and EPA + DHA from FDLs were observed (all *p* < 0.001; Table 4).

The categorical adequacy analysis of dietary fat intake is provided in Supplementary Table S2. Briefly, at T2, the proportion of participants meeting the EPA + DHA adequacy threshold increased in both groups and reached 100% in the IG. In the IG, the proportion of participants within the recommended ranges increased for MUFA and PUFA intake but decreased for total fat and SFA intake.

### 3.3. Intervention and Changes in Inflammatory Status

For circulating cytokines, IL-8 increased within the IG (*p* = 0.015), with a significant between-group difference in change (*p* = 0.030; Table 5). However, the adjusted effect did not reach statistical significance (adj. fold-change (IG/CG): 2.50; 95% CI: 0.74 to 8.48; *p* > 0.05). IL-10 increased within the IG (*p* = 0.024) and ANCOVA indicated a significant adjusted intervention effect (adj. fold-change (IG/CG): 5.07; 95% CI: 1.32 to 19.48; *p* = 0.019), while the between-group comparison of change was not significant (*p* > 0.05). Threshold-adjusted effects were observed for IL-1β (adj. fold-change (IG/CG): 2.91 (95% CI 1.00 to 8.45), *p* = 0.050) and TNF-α (adj. fold-change (IG/CG): 2.71 (95% CI 1.00 to 7.38), *p* = 0.051), with a similar threshold pattern for IL-6 (adj. fold-change (IG/CG): 4.43 (95% CI 0.96 to 20.54), *p* = 0.057).

No significant within-group changes in fecal CAL were observed from T0 to T2 in either the CG or IG (both *p* > 0.05), and changes did not differ between groups (*p* > 0.05). ANCOVA showed no adjusted intervention effect (adj. fold-change (IG/CG): 0.86; 95% CI: 0.50 to 1.47; *p* > 0.05).

For mitogen-stimulated responses, (PHA), IL-1 (PHA) and IL-6 (PHA) decreased within the CG (both *p* = 0.003), and IL-8 (PHA) also decreased within the CG (*p* = 0.011; Table 6). In the IG, no significant within-group changes were observed for any PHA-stimulated cytokine (all *p* > 0.05). Between-group differences in change were significant for IL-6 (PHA) (*p* = 0.025) and IL-8 (PHA) (*p* = 0.017). However, ANCOVA showed no adjusted intervention effects for IL-6 (PHA) (adj. fold-change (IG/CG): 0.98; 95% CI: 0.29 to 3.36; *p* > 0.05) or IL-8 (PHA) (1.18; 95% CI: 0.67 to 2.07; *p* > 0.05). No significant effects were observed for IL-10 (PHA) or TNF-α (PHA) in within-group, between-group, or ANCOVA analyses (all *p* > 0.05).

### 3.4. Associations Between Changes in Diet and Inflammatory Status

**Figure 2:**
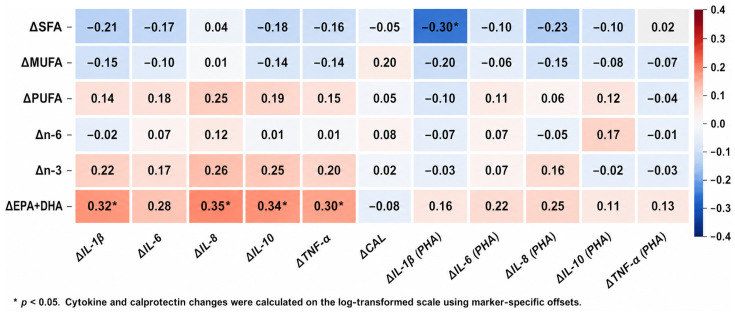
Heatmap of Spearman correlations between changes in dietary variables and changes in inflammatory biomarkers. The heatmap presents Spearman’s rank correlation coefficients (ρ) between changes from (T0) baseline to week 12 (T2). Changes were calculated as T2-T0. Variables were analyzed on the natural log scale using ln(value + offset). Offsets used in ln(value + offset) were as follows: IL-1β offset = 1; IL-8 offset = 106; IL-10 offset = 1; TNF-α offset = 1.5; IL-1β (PHA) offset = 10. IL-1β, interleukin-1 beta; IL-6, interleukin-6; IL-8, interleukin-8; IL-10, interleukin-10; TNF-α, tumor necrosis factor alpha; MUFA, monounsaturated fatty acid; PUFA, polyunsaturated fatty acid; SFA, saturated fatty acid; *n*-6, Omega-6 fatty acid; *n*-3, Omega-3 fatty acid; EPA, eicosapentaenoic acid; DHA, docosahexaenoic acid; CAL, fecal calprotectin; PHA, phytohemagglutinin; * *p*-value < 0.05.

Exploratory associations between changes in dietary variables and changes in inflammatory biomarkers are summarized in Figure 2. The strongest positive correlation was observed between ΔEPA + DHA and ΔIL-8 (ρ = 0.35, *p* = 0.016), followed by positive correlations with ΔIL-10 (ρ = 0.34, *p* = 0.022), ΔIL-1 β (ρ = 0.32, *p* = 0.030), and ΔTNF-α (ρ = 0.30, *p* = 0.042). In contrast, the strongest inverse correlation was observed between ΔSFA and ΔIL-1 (PHA) (ρ = −0.30, *p* = 0.043).

**Figure 3:**
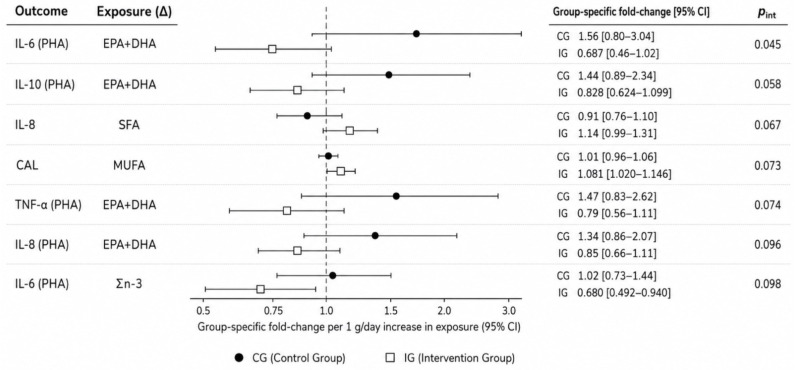
Forest plot of group-specific dietary exposure–biomarker associations. Forest plot showing group-specific fold-changes with 95% confidence intervals for associations between changes in dietary exposures and biomarker changes from baseline to week 12. Values greater than 1 indicate a positive association, whereas values below 1 indicate an inverse association. The vertical reference line represents no association, fold-change = 1. Interaction *p*-values correspond to the Group × ΔExposure term. CG, control group; IG, intervention group; PHA, phytohemagglutinin; CAL, fecal calprotectin; SFA, saturated fatty acids; MUFA, monounsaturated fatty acids; EPA, eicosapentaenoic acid; DHA, docosahexaenoic acid; Σ*n*-3, total Omega-3 fatty acids; p_int, *p*-value for the interaction; CI, confidence interval.

Group-specific associations from the interaction models are presented in Figure 3. The clearest exploratory interaction was observed for IL-6 (PHA) × ΔEPA + DHA (*p* = 0.045; fold-change: 0.44 [0.20–0.96]), indicating that the association between changes in EPA + DHA intake and changes in stimulated IL-6 differed between groups. Suggestive trends toward interaction were also observed for EPA + DHA with IL-10 (PHA) (*p* = 0.058), TNF-α (PHA) (*p* = 0.074) and IL-8 (PHA) (*p* = 0.096), as well as for ΔSFA with IL-8 (*p* = 0.067), ΔMUFA with CAL (*p* = 0.073), and Δ*n*-3 with IL-6 (PHA) (*p* = 0.098). Where interactions were at the threshold, group-specific estimates suggested heterogeneity for selected associations (e.g., ΔMUFA-CAL: CG fold-change 1.01 [0.96–1.06], *p* = 0.835 vs. IG fold-change 1.081 [1.020–1.146], *p* = 0.011; Δ*n*-3–IL-6 (PHA): CG fold-change 1.02 [0.73–1.44], *p* = 0.903 vs. IG fold-change 0.680 [0.492–0.940], *p* = 0.024).

### 3.5. Changes in Clinical Outcomes

#### 3.5.1. Health-Related Quality of Life Assessment (EHP-30)

In the CG, EHP-30 Pain scores decreased from T0 to T2 (*p* = 0.016), and Emotional well-being improved (*p* = 0.033), while no significant within-group changes were observed for Control and powerlessness, Social support, or Self-image (all *p* > 0.05) (Table 7). In the IG, EHP-30 Pain scores decreased from T0 to T2 (*p* = 0.002) whereas changes in Control and powerlessness, Emotional well-being, Social support, and Self-image (all *p* > 0.05) were not significant. Between-group comparisons of change (Δ) were not significant across any EHP-30 domain (all *p* > 0.05). Consistently, ANCOVA showed no significant adjusted intervention effects for EHP-30 Pain, Control and powerlessness, Emotional well-being, Social support, and Self-image (all *p* > 0.05).

#### 3.5.2. Changes in Pain Intensity Assessment (VAS)

Pain intensity, as assessed using the VAS, did not change significantly in the CG from T0 to T2 (4.9 ± 2.2; median [IQR]: 4.0 [4.0; 6.0] vs. 4.5 ± 2.3; 4.0 [3.5; 6.0]; *p* > 0.05). In the IG, VAS pain intensity decreased significantly from T0 to T2 (5.0 ± 2.1; 5.0 [4.0; 6.0] vs. 4.3 ± 2.1; 4.0 [3.5; 5.5]; *p* = 0.011). However, the between-group comparison of change scores was not statistically significant (*p* > 0.05), and ANCOVA showed no significant adjusted between-group effect (adjusted effect [IG-CG]: −0.37; 95% CI: −1.16 to 0.42; *p* > 0.05).

For the ≥1-point threshold, the responder rate was higher in the IG than in the CG (73.9% vs. 34.8%), indicating a significantly greater probability of response in the IG (RR [IG vs. CG] = 2.13, 95% CI: 1.16; 3.91; *p* = 0.015). This finding remained after adjustment for baseline pain intensity (adjusted RR [IG vs. CG] = 2.11, 95% CI: 1.16; 3.89; *p* = 0.014). For the ≥2-point threshold, responder rates were similar between groups, with no significant between-group difference (*p* > 0.05); adjustment for baseline pain did not change these findings (*p* > 0.05).

## 4. Discussion

FDL supplementation with a diet based on the Healthy Eating Plate was associated with a selective immunological response in women with endometriosis, mainly reflected by a significant adjusted between-group effect for serum IL-10. Other inflammatory biomarkers, including IL-1β, IL-6, IL-8, TNF-α, and fecal CAL, did not demonstrate consistent significant between-group effects. Similarly, although within-group changes in pain-related outcomes were observed, adjusted between-group analyses did not confirm a significant effect on VAS pain intensity or EHP-30 domains. However, a ≥1-point reduction in VAS pain intensity was noticed more frequently in the IG than in the CG. This finding may suggest a potential clinically relevant signal, but it should be interpreted cautiously.

This interpretation is consistent with evidence indicating that increased EPA/DHA exposure does not uniformly reduce systemic cytokines (e.g., IL-6) across settings [38]. Moreover, the direction and magnitude of cytokine changes following *n*-3 PUFA supplementation appear to depend on clinical context, baseline inflammatory phenotype, dose, duration of exposure, and the immune endpoint assessed. Although evidence from clinically distinct populations should be extrapolated with caution, previous studies have shown that *n*-3 PUFA supplementation may produce heterogeneous cytokine responses rather than uniform cytokine suppression [39]. Collectively, these observations support the view that *n*-3 PUFA-related immunological effects are context-dependent and may reflect immune rebalancing rather than generalized immunosuppression. In line with this, fish oil supplementation has been reported to increase IL-10 and shift cytokine patterns toward a more regulatory profile in some populations [40]. Furthermore, Sijben and Calder noted that in healthy adults, long-chain *n*-3 PUFA supplementation most often shows no consistent effects on ex vivo lymphocyte proliferation or cytokine production, and when changes occur, their direction is variable and more likely at higher intakes (≥2 g EPA + DHA/day), whereas effects are more frequently observed in chronic inflammatory states [41]. Recent omics-enabled evidence also indicates marked interindividual variability and engagement of multiple immunometabolism pathways, suggesting that detectable cytokine shifts depend on baseline phenotype, and endpoint selection [42]. FDL interventions may also act through immune cell functional pathways that are not captured by basal circulating cytokines. In a trial in healthy volunteers, FDLs enhanced natural killer (NK) cell cytotoxicity and increased interferon gamma (IFN-γ) synthesis, with effects linked to free fatty acid receptors (FFAR1/FFAR4) on lymphocytes. This highlights the value of incorporating immune cell phenotyping and functional assays (e.g., NK cytotoxicity, IFN-γ production, FFAR-related signaling) alongside cytokines in future endometriosis trials to better define mechanisms and sources of interindividual variability [25].

IL-10 is a central regulatory cytokine that limits excessive immune activation and supports immune homeostasis in chronic inflammatory conditions [43,44]. Although the IL-10 response is compatible with resolution-oriented biology, this inference remains indirect because we did not quantify specialized pro-resolving mediators (SPMs) or immune cell phenotypes. Prior interventions have reported that sustained *n*-3 exposure can increase circulating SPMs (e.g., resolvin D1) alongside increases in IL-10, and experimental work suggests that resolvin D1 may promote IL-10 production. In a 12-week trial, Polus et al. reported increased plasma resolvin D1 accompanied by higher IL-10 following dietary guidance plus 1.8 g/day *n*-3, supporting the plausibility that sustained *n*-3 exposure may engage regulatory cytokine signaling [45]. Nevertheless, the present study did not quantify SPMs or immune cell phenotypes. Notably, the intervention increased not only EPA/DHA but also other constituents of fish-derived lipids, including squalene and alkylglycerols. Experimental data suggest that squalene can modulate macrophage responses and NF-κB-related pathways and may influence anti-inflammatory outputs, including IL-10 [46]. Alkylglycerols have been linked to ether lipid remodeling and changes in inflammatory mediator profiles, providing an additional plausible pathway through which FDLs may affect regulatory immune signaling [47]. Collectively, these findings support a cautious interpretation that increased IL-10 may reflect regulatory adaptation even when changes in canonical pro-inflammatory cytokines are heterogeneous.

To capture complementary dimensions of immune activity, in our study, cytokines were assessed both at rest and after PHA stimulation, which provides an index of immune cell activation potential. PHA is a classical T-cell mitogen used in ex vivo assays to probe functional cytokine responsiveness; nonetheless, this readout should be interpreted as a functional measure rather than a direct surrogate of the peritoneal/lesional milieu, and its meaning depends on the stimulation platform (e.g., PBMC based vs. whole blood assays). For PHA-stimulated cytokines, the within-group decreases in IL-1β (PHA), IL-6 (PHA), and IL-8 (PHA) in the CG are consistent with the notion that dietary optimization alone may attenuate mitogen-induced immune responsiveness. This pattern is biologically plausible because improvements in overall diet quality including higher intake of fiber-rich plant foods and antioxidant or microbiota-supporting nutrients have been linked to immune homeostasis and reduced pro-inflammatory signaling, potentially mediated by improved metabolic status and SCFA-related immunoregulation [7,48,49,50,51,52,53,54]. Although adjusted models did not confirm a statistically significant intervention effect for stimulated cytokines, the directionality in the CG is compatible with a higher activation threshold under ex vivo stimulation. By contrast, the absence of comparable decreases in the IG may indicate a different immunomodulatory profile associated with high *n*-3 PUFA exposure or accompanying bioactive lipids, potentially reflecting immune adaptation/remodeling rather than uniform suppression of cytokine output.

In interaction models, we observed nominal evidence of group-specific effect modification for ΔEPA + DHA on PHA-stimulated IL-6, with additional threshold trends for TNF-α and IL-10. Greater increases in ΔEPA + DHA were directionally associated with lower stimulated IL-6, TNF-α and IL-10 in the IG, whereas the corresponding associations in the CG were positive and not statistically significant. Although exploratory, this pattern is consistent with selective immunomodulation affecting functional cytokine responsiveness under standardized stimulation rather than uniformly altering resting circulating cytokines. This interpretation aligns with the proposition that long-chain *n*-3 PUFA effects may be more readily detected in functional immune outcomes than in basal systemic cytokines [41] and with emerging evidence that responses to *n*-3 exposure are context-dependent and highly variable between individuals, likely influenced by baseline phenotype and clinical setting [42].

The cytokine pattern observed in the present study did not indicate a uniform anti-inflammatory response to FDL supplementation. Although TNF-α values above the assay-specific laboratory reference range may indicate an inflammatory state [55], this observation should be interpreted in the context of the chronic inflammatory nature of endometriosis, in which elevated serum concentrations of IL-1β, IL-6, and TNF-α have previously been reported [9]. Therefore, the cytokine profile observed in this study should be interpreted in the context of the pre-existing inflammatory milieu characteristic of this disease, which may also influence the individual response to FDL supplementation. Specifically, the IG showed an increase in the regulatory cytokine IL-10, accompanied by an increase in IL-8 and borderline signals for IL-1β and TNF-α, with a similar tendency for IL-6. Individual cytokine trajectories suggested the heterogeneity of response; however, these observations are descriptive and are not based on a predefined responder analysis. Therefore, they should be interpreted as evidence of interindividual variability in cytokine trajectories rather than as proof of a generalized inflammation-reducing effect of FDL supplementation.

Such heterogeneity is biologically plausible, as endometriosis is characterized by immune dysregulation and compartmentalized inflammatory activity within the peritoneal and lesional microenvironment, which may not be fully reflected by circulating cytokine concentrations [12,56]. Endometriotic lesions develop within an estrogen-dependent inflammatory milieu in which ectopic endometrium-like tissue, mesothelial cells, macrophages, lymphocytes, cytokines, chemokines, growth factors, nerve fibers, and lipid mediators interact locally [12,56,57,58]. In particular, peritoneal macrophages may accumulate in women with endometriosis but show impaired phagocytic clearance, while producing inflammatory, angiogenic, fibrotic, and neurotrophic mediators that may support lesion persistence, neuroinflammation, angiogenesis, innervation, and pain sensitization [12,56,59]. Accordingly, substantial between-study variability has been reported, and single circulating cytokines appear to have limited standalone clinical utility as biomarkers of endometriosis [59]. In this context, the IL-10 increase may be interpreted as a selective immunomodulatory response rather than broad cytokine suppression. However, the direction of cytokine responses was not uniformly anti-inflammatory. The positive exploratory correlations between changes in EPA + DHA intake and changes in IL-1β, IL-8, and TNF-α were unexpected and should not be interpreted as evidence of a pro-inflammatory effect of *n*-3 fatty acids. Fish-derived lipids and *n*-3 fatty acids, especially EPA and DHA, are generally considered immunomodulatory fatty acids that may influence inflammatory processes through eicosanoid production, cytokine signaling, leukocyte function, and the generation of specialized pro-resolving mediators, including resolvins, protectins, and maresins [28,60]. Several alternative explanations should therefore be considered. First, reverse causality is possible, as participants with greater inflammatory activity, symptom burden, or stronger motivation to modify their diet may have reported greater increases in EPA + DHA intake. Second, EPA + DHA intake was estimated from self-reported dietary records, which are vulnerable to measurement error, underreporting, and changes in reporting accuracy during dietary interventions [61,62]. Third, ex vivo PHA-stimulated cytokine responses reflect immune cell activation potential rather than direct inflammatory activity within the peritoneal or lesional microenvironment. Such responses may vary substantially between individuals and may not parallel unstimulated circulating cytokine concentrations [63]. Fourth, the 12-week intervention may have been too short to capture stable changes in complex immune, neuroinflammatory, and pain-related pathways in endometriosis, particularly in the absence of objective fatty acid incorporation markers. Overall, these observations support the view that *n*-3 PUFA-related immunological effects may be context-dependent and influenced by baseline phenotype, inflammatory status, endpoint selection, and immune cell functional responses [64,65]. Therefore, the observed findings cannot be attributed solely to *n*-3 fatty acids and should be considered exploratory and hypothesis-generating.

Fecal CAL, a neutrophil-derived marker of intestinal inflammation reflecting mucosal inflammatory activity [66], remained unchanged during the intervention, suggesting no clear short-term effect on this proxy of intestinal neutrophil activity. In addition, fecal calprotectin shows meaningful within-person variability across collections, which may reduce sensitivity to detect modest changes from single pre- or post-intervention measurements [67].

Although the responder analysis indicated that a ≥1-point reduction in VAS pain intensity occurred more frequently in the IG than in the CG, adjusted mean-based between-group analyses did not confirm a significant effect on VAS pain intensity or EHP-30 domains. Therefore, this finding may represent a potential exploratory clinical signal, but it should not be interpreted as definitive evidence of intervention efficacy. This pattern is consistent with observational evidence showing that many women report diet-related symptom benefits despite variable alignment with objective outcomes [68], supporting the rationale for individualized dietary approaches. A cross-sectional study has also associated adherence to an “endometriosis diet” with improved QoL, suggesting potential longer-term benefits [69]. In addition, randomized feeding trials indicate that targeted dietary strategies such as a low-FODMAP diet can improve gastrointestinal symptoms and QoL over 4 weeks [70,71]. Collectively, these findings suggest that clinical effects may depend on intervention duration and phenotype-specific targeting. Similarly, Nodler et al. reported that six months of fish oil supplementation did not yield clinically meaningful pain reductions versus placebo in adolescents and young women with endometriosis, underscoring placebo effects and the difficulty of attributing symptom changes to supplementation alone [72]. A meta-analysis similarly reported no significant improvement in endometriosis-related symptoms with *n*-3 fatty acids across outcomes [73], although benefits have been observed in related pain contexts such as dysmenorrhea [74] and within complex nutraceutical formulations [75]. Mediterranean diet adherence has also been associated with pain reduction over 3–6 months [76], reinforcing the potential importance of longer-term and targeted dietary frameworks.

Overall, the present findings suggest that short-term FDL exposure may increase the likelihood of modest symptom improvement in a subset of women and may engage regulatory immune signaling, as reflected by the increase in IL-10. However, consistent reductions in circulating pro-inflammatory cytokines were not detected, and improvements in VAS and EHP-30 were modest at the group level but heterogeneous across participants. These patterns, together with limited mean between-group differences, underscore the need for larger, adequately powered trials with longer follow-up, objective adherence measures, repeated biomarker assessments including stool markers, and mechanistic endpoints to clarify pathways and identify responders most likely to benefit.

The strengths of this study include well-matched groups at baseline, the use of validated instruments for symptom and QoL assessment, and evaluation of both metabolic and immunological parameters. These design features allowed for a comprehensive evaluation of clinical, metabolic, and immunological outcomes.

Limitations include the relatively short intervention duration and modest sample size, which limited the power to detect small-to-moderate effects on clinical endpoints such as pain and QoL. Dietary intake was primarily assessed using self-reported methods and is therefore prone to recall and reporting bias, as well as random measurement error. Although dietary analyses demonstrated clear separation in marine lipid exposure, the study lacked objective incorporation/adherence measures that would strengthen causal inference, such as the erythrocyte *n*-3 index (or other blood fatty acid composition biomarkers) or biochemical markers specifically reflecting the non-PUFA components of the preparation (e.g., squalene-related or ether-alkylglycerol-derived signatures). Accordingly, systemic inflammatory findings should be interpreted cautiously, especially in endometriosis, where inflammation is known to be compartmentalized within the peritoneal cavity and lesions. Consequently, circulating cytokine profiles may be heterogeneous across phenotypes and have limited diagnostic or monitoring utility when interpreted as single markers [3,8,12,59]. This underscores the value of controlled interventions with clinically relevant endpoints and cautious interpretation of systemic biomarker changes.

Importantly, the intervention did not demonstrate significant adjusted between-group effects for most inflammatory biomarkers, fecal calprotectin, VAS pain intensity, or EHP-30 domain scores. Therefore, the observed within-group changes and exploratory associations should not be interpreted as evidence of intervention efficacy. In addition, multiple secondary and exploratory analyses were performed, including correlation and interaction analyses, without formal correction for multiple comparisons. Consequently, nominally significant findings should be interpreted cautiously and considered hypothesis-generating.

From a clinical perspective, the present findings support the relevance of further investigating FDL supplementation, when added to a Healthy Eating Plate-based dietary intervention, as a potential supportive component of multidisciplinary endometriosis care. However, these results should not be interpreted as evidence of clinical efficacy or as indicating that FDL supplementation or nutritional intervention can replace standard medical or surgical management. Future studies should include larger sample sizes, longer follow-up periods, stricter control of previous and ongoing treatments, and predefined inflammatory phenotypes. Further research should also evaluate whether fecal calprotectin and selected cytokine profiles may help identify subgroups of patients who are more likely to benefit from FDL supplementation combined with a Healthy Eating Plate-based dietary intervention. Furthermore, future studies should include repeated immunological measurements, the use of objective markers of fatty acid status, such as the *n*-3 erythrocyte index, and peritoneal immune profiling, macrophage phenotyping, lipid mediator analysis, and functional immune cell assays to better define the underlying mechanisms and sources of interindividual variability.

## 5. Conclusions

In this exploratory controlled intervention study, supplementation of a Healthy Eating Plate-based diet with FDLs did not demonstrate clear superiority over diet alone for most inflammatory biomarkers, fecal calprotectin, mean VAS pain intensity, or EHP-30 quality-of-life domains after 12 weeks. A significant adjusted between-group effect was observed for IL-10, as well as a greater likelihood of reducing pain intensity on the VAS in the IG. Selected exploratory associations were also identified between changes in dietary fatty acid intake and cytokine changes. However, the investigated formulation was not based on isolated *n*-3 fatty acids, but on a biologically derived fish–lipid mixture with a complex lipid profile. The obtained results, indicating possible changes in immunomodulatory signals and those related to pain symptoms as an effect of FDL supplementation, should be interpreted with caution and confirmed in larger studies with longer follow-up or intervention periods, taking into account the potential mechanisms of biological response.

## Figures and Tables

**Table 1:** Characteristics of the study participants.

Parameters	CG	IG	*p*-Value
Age	34.4	34.7	0.914
Time since diagnosis of endometriosis (%)			1.00
Up to 12 months	38	39	
More than 12 months	62	61	
WC (cm)	82.4 ± 12.6	76.7 ± 10.6	0.082
WHR	0.80 ± 0.07	0.77 ± 0.08	0.187
WHtR	0.49 ± 0.07	0.46 ± 0.05	0.065
Body fat (%)	27.3 ± 7.4	23.3 ± 7.5	0.074
Physical activity (≥3 times/week)—high (%)	52	56	1.00
Physical activity—number of days/week (mean ± SD)	2.3 ± 1.1	2.8 ± 1.3	0.166
General self-rated well-being (%)			0.921
Poor	4	9	
Fairly good	30	30	
Good	61	52	
Very good	4	9	

Data are presented as *n* (%) or mean; CG, control group; IG, intervention group; WC, waist circumference; WHR, waist-to-hip ratio; WHtR, waist-to-height ratio; general self-rated well-being was assessed at baseline using a single self-report item from the screening questionnaire with four response categories: poor, fairly good, good, and very good; for the results of the *t*-Student test or the U-Mann–Whitney test; *p*-value < 0.05.

**Table 2:** Characteristics of energy and macronutrients (mean ± SD; median [IQR]).

Marker	CG T0	CG T2	*p*-Value Within (CG)	IG T0	IG T2	*p*-Value Within (IG)	*p*-Value Between (Δ)	ANCOVA adj. Effect (IG − CG) [95% CI]	*p*-Value ANCOVA
Energy (kcal/day)	1768 ± 263; 1756 [1663; 1953]	1902 ± 185; 1900 [1759; 2005]	0.050	1763 ± 352; 1772 [1502; 1950]	1992 ± 237; 2050 [1700; 2150]	0.013	0.185	92.7 [−6.35; 191.8]	0.066
Protein (% energy)	20.5 ± 3.1; 20.6 [18.5; 22.1]	20.7 ± 2.0; 20.6 [19.4; 22.2]	0.590	18.2 ± 3.4; 18.7 [16.3; 20.6]	19.0 ± 2.0; 19.0 [17.7; 20.0]	0.291	0.869	−0.80 [−1.75; 0.15]	0.098
Carbohydrate (% energy)	43.1 ± 7.3; 43.8 [41.75; 46.6]	43.1 ± 5.5; 44.2 [39.7; 47.3]	0.990	43.1 ± 7.0; 40.8 [37.5; 49.9]	35.9 ± 4.9; 36.3 [31.7; 39.0]	<0.001	<0.001	−7.21 [−9.71; −4.71]	<0.001
Total fat (% energy)	36.4 ± 5.61; 35.4 [32.6; 38.0]	36.1 ± 4.9; 35.0 [32.9; 40.2]	0.859	38.7 ± 6.5; 38.9 [33.3; 42.9]	45.1 ± 4.8; 45.8 [41.7; 48.3]	<0.001	<0.001	7.86 [5.43; 10.29]	<0.001

Data are presented as mean ± SD and median [IQR]; SD, standard deviation; IQR, interquartile range; T0, baseline; T2, end of intervention; CG, control group; IG, intervention group; within-group comparisons were performed using paired *t*-test or Wilcoxon signed-rank test; between-group Δ(T2 − T0) comparisons were performed using independent *t*-test or Mann–Whitney U test; ANCOVA: T2~group + T0; effect reported as (IG − CG) with 95% CI; CI, confidence interval; Δ, change from baseline to week 12; *p*-value < 0.05.

**Table 3:** Changes in fatty acid intake between T0 and T2 (mean ± SD; median [IQR]).

Marker	CG T0	CG T2	*p*-Value Within (CG)	IG T0	IG T2	*p*-Value Within (IG)	*p*-Value Between (Δ)	ANCOVA adj. Effect (IG − CG) [95% CI]	*p*-Value ANCOVA
SFA (g/day)	19.52 ± 7.47; 19.00 [15.70; 20.30]	20.38 ± 5.07; 20.10 [18.00; 21.05]	0.144	22.68 ± 8.43; 20.40 [18.35; 23.95]	24.37 ± 5.25; 23.71 [21.35; 26.05]	0.111	0.311	2.83 [0.22; 5.43]	0.034
MUFA (g/day)	22.69 ± 7.29; 21.90 [18.35; 24.30]	27.83 ± 7.02; 28.00 [25.10; 31.30]	0.011	25.67 ± 9.73; 24.20 [19.60; 31.30]	32.02 ± 7.85; 30.70 [27.00; 36.25]	<0.001	0.628	3.01 [−1.04; 7.06]	0.141
PUFA (g/day)	11.75 ± 5.07; 11.20 [8.85; 12.15)	14.41 ± 5.98; 14.70 [9.30; 16.40)	0.052	12.98 ± 6.42; 11.90 [9.05; 16.25]	23.48 ± 5.16; 22.12 [19.00; 25.50]	<0.001	<0.001	8.60 [5.50; 11.72]	<0.001
Σ *n*-6 fatty acids (g/day)	8.49 ± 3.30; 8.30 [6.40; 8.70]	10.15 ± 4.68; 9.55 [6.95; 12.40]	0.145	9.63 ± 4.67; 9.40 [6.65; 11.40]	9.66 ± 4.38; 8.80 [7.60; 10.00]	0.983	0.354	−0.86 [−3.50; 1.77]	0.512
Σ *n*-3 fatty acids (g/day)	3.27 ± 2.22; 2.90 [1.95; 3.45]	4.26 ± 1.89; 4.10 [3.15; 4.65]	0.006	3.35 ± 2.40; 2.45 [1.90; 3.95]	13.82 ± 2.22; 13.03 [12.16; 15.69]	<0.001	<0.001	9.53 [8.41; 10.65]	<0.001
EPA + DHA (g/day)	1.49 ± 1.17; 1.00 [0.50; 2.40]	2.10 ± 1.08; 1.90 [1.50; 2.50]	0.021	1.14 ± 0.90; 1.20 [0.20; 1.70]	9.82 ± 1.74; 9.42 [8.74; 10.60]	<0.001	<0.001	7.81 [6.93; 8.68]	<0.001

Data are presented as mean ± SD and median [IQR]; SD, standard deviation; IQR, interquartile range; T0, baseline; T2, end of intervention; CG, control group; IG, intervention group; SFA, saturated fatty acids; MUFA, monounsaturated fatty acid; PUFA, polyunsaturated fatty acid; *n*-6, Omega-6 fatty acid; *n*-3, Omega-3 fatty acid; EPA, eicosapentaenoic acid; DHA, docosahexaenoic acid. Within-group comparisons were performed using paired *t*-test or Wilcoxon signed-rank test; between-group Δ(T2 − T0) comparisons were performed using independent *t*-test or Mann–Whitney U test; ANCOVA: T2~group + T0; effect reported as (IG − CG) with 95% CI; CI, confidence interval; Σ denotes the sum; Δ, change from baseline to week 12; *p*-value < 0.05.

**Table 4:** Mean values of fatty acid intake from fish oil in the intervention group (IG) (at T2).

Component Group	Component	IG T2 (Mean ± SD Median [IQR])	*p*-Value Within (IG)
Complex lipids	Alkylglycerols	2.40 ± 0.57; 2.34 [2.08; 2.68]	<0.001
	Squalene	2.32 ± 0.48; 2.20 [2.01; 2.54]	<0.001
*n*-3 fatty acids	EPA + DHA	7.74 ± 1.40; 7.50 [6.58; 8.50]	<0.001

Data are presented as mean ± SD; median [IQR]; SD, standard deviation; IQR, interquartile range; T0, baseline; T2, end of intervention; IG, intervention group; *n*-3 fatty acid, Omega-3 fatty acid; EPA, eicosapentaenoic acid; DHA, docosahexaenoic acid. For Squalene and Alkylglycerols, baseline (T0) values were 0 (no supplementation prior to T0); EPA + DHA reflects total intake and is presented for both T0 and T2.

**Table 5:** Changes in circulating cytokines and fecal calprotectin between T0 and T2 (mean ± SD; median [IQR]).

Marker	CG T0	CG T2	*p*-Value Within (CG)	IG T0	IG T2	*p*-Value Within (IG)	*p*-Value Between (Δ)	ANCOVA adj. Fold-Change (IG/CG) [95% CI]	*p*-Value ANCOVA
**IL-1 β (pg/mL)**	153 ± 210; 71 [30; 216]	277 ± 622; 27.00 [10.50; 132.50]	0.855	133 ± 193; 56 [18.5; 128]	301 ± 302; 193 [67; 472]	0.089 †	0.170	2.91 [1.00; 8.45]	0.050
**IL-6 (pg/mL)**	7740 ± 15,966; 2193 [607; 4758]	40,235 ± 124,987; 494 [200; 12,871]	0.501	9783 ± 18,823; 1907 [335; 7284]	46,813 ± 89,280; 9051 [2991; 33,015]	0.092 †	0.455	4.43 [0.96; 20.54]	0.057
**IL-8 (ng/mL)**	133± 157; 107 [33.5; 149]	159± 309; 17.9 [9.0; 182]	0.540	92.5 ± 85.4; 76.8 [20.7; 145]	307± 394 179 [37.3; 336]	0.015	0.030	2.50 [0.74; 8.48]	0.138
**IL-10 (pg/mL)**	32.8 ± 44.3; 14.0 [4.5; 37.0]	157 ± 576; 5.0 [0.0; 63.0]	0.615	32.5 ± 51.5; 12.0 [1.5; 50.0]	265 ± 508; 41 [17.5; 153]	0.024	0.127	5.07 [1.32; 19.48]	0.019
**TNF-α (pg/mL)**	149 ± 221; 104 [26; 177]	262 ± 619; 22 [13.5; 135]	0.303	136 ± 217; 76 [25; 155]	270 ± 328; 168 [102; 243]	0.086 †	0.132	2.71 [1.00; 7.38]	0.051
**CAL (µg/g)**	14.1 ± 16.3; 7.3 [3.5; 19.8]	10.1 ± 8.4; 7.5 [4.0; 11.3]	0.766	9.1 ± 9.9; 7.2 [3.4; 11.0]	9.0 ± 9.2; 7.3 [3.3; 12.3]	0.861	0.652	0.86 [0.50; 1.47]	0.593

Data are presented as mean ± SD and median [IQR]; SD, standard deviation; IQR, interquartile range; T0, baseline; T2, end of intervention; CG, control group; IG, intervention group; IL-1β, interleukin-1 beta; IL-6, interleukin-6; IL-8, interleukin-8; IL-10, interleukin-10; TNF-α, tumor necrosis factor alpha; CAL, calprotectin. Within-group comparisons were performed using paired *t*-test or Wilcoxon signed-rank test; between-group Δ(T2 − T0) comparisons were performed using independent *t*-test or Mann–Whitney *U* test. ANCOVA: T2~group + T0; ANCOVA was performed on the log-transformed scale using ln(value + offset): ln(T2 + offset)~group + ln(T0 + offset); offsets used in ln(value + offset): IL-1β offset = 1; IL-6 offset = 11; IL-8 offset = 106; IL-10 offset = 1; TNF-α offset = 1.5; CAL offset = 0.2. Adjusted effects are back-transformed and reported as effect ratios (fold-changes) for IG vs. CG (IG/CG) with 95% CI; CI, confidence interval; Δ, change from baseline to week 12; † borderline significance was defined as 0.05 ≤ *p* < 0.10; *p*-value < 0.05.

**Table 6:** Changes in PHA-stimulated cytokine production between T0 and T2 (mean ± SD; median [IQR]).

Marker	CG T0	CG T2	*p*-Value Within (CG)	IG T0	IG T2	*p*-Value Within (IG)	*p*-Value Between (Δ)	ANCOVA adj. Fold-Change (IG/CG) [95% CI]	*p*-Value ANCOVA
IL-1β (PHA) (pg/mL)	1912 ± 1007; 1535 [1166; 2717]	1280± 1483; 745 [393; 1487]	0.003	1400 ± 836; 1266 [835; 1562]	1463 ± 1484; 1301 [528; 1645]	0.370	0.091 †	1.37 [0.66; 2.86]	0.391
IL-6 (PHA) (ng/mL)	167 ± 98; 126 [109; 196]	106 ± 117; 79 [35.9; 140]	0.003	109 ± 57; 104 [666; 149]	121 ± 111; 90 [554; 165]	0.667	0.025	0.98 [0.29; 3.36]	0.978
IL-8 (PHA) (ng/mL)	369 ± 245; 290 [207; 510]	187 ± 166; 160 [92; 176]	0.011	232 ± 148; 164 [145; 260]	296 ± 260; 151 [122; 483]	0.378	0.035	1.18 [0.67; 2.07]	0.558
IL-10 (PHA) (pg/mL)	744 ± 305; 649 [479; 984]	558 ± 378; 436 [303; 747]	0.092 †	708 ± 310; 688 [474; 979]	626 ± 457; 607 [309; 782]	0.467	0.503	0.89 [0.40; 2.00]	0.780
TNF-α (PHA) (pg/mL)	1706 ± 1111; 1235 [1093; 1765]	1511 ± 1574; 1067 [615; 1946]	0.294	1363 ± 836; 1130 [822; 1637]	1338 ± 1106; 1098 [732; 1720]	0.930	0.489	0.71 [0.29; 1.76]	0.452

Data are presented as mean ± SD and median [IQR]; SD, standard deviation; IQR, interquartile range; T0, baseline; T2, end of intervention; CG, control group; IG, intervention group; IL-1β, interleukin-1 beta; IL-6, interleukin-6; IL-8, interleukin-8; IL-10, interleukin-10; TNF-α, tumor necrosis factor alpha; PHA, phytohemagglutinin. Within-group comparisons were performed using paired *t*-test or Wilcoxon signed-rank test; between-group Δ(T2 − T0) comparisons were performed using independent *t*-test or Mann–Whitney U test; ANCOVA: T2~group + T0; ANCOVA was performed on the log-transformed scale using ln(value + offset): ln(T2 + offset)~group + ln(T0 + offset); offset used in ln(value + offset) (PHA): IL-1β (PHA) offset = 10; IL-6 (PHA) offset = 36.5; IL-8 (PHA) offset = 5500; IL-10 (PHA) offset = 2; TNF-α (PHA) offset = 3. Adjusted effects are back-transformed and reported as effect ratios (fold-changes) for IG/CG with 95% CI; CI, confidence interval; Δ, change from baseline to week 12; † borderline significance was defined as 0.05 ≤ *p* < 0.10; *p*-value < 0.05.

**Table 7:** Changes in EHP-30 domains at T0 and T2 (mean ± SD; median [IQR]).

Domain EHP-30	CG T0	CG T2	*p*-Value Within (CG)	IG T0	IG T2	*p*-Value Within (IG)	*p*-Value Between (Δ)	ANCOVA adj. Effect (IG-CG) [95% CI]	*p*-Value ANCOVA
**Pain**	56.5 ± 18.8; 54.5 [45.5; 65.9]	52.9 ± 20.4; 54.5 [40.9; 64.8]	0.016	57.0 ± 16.7; 59.1 [47.7; 68.2]	50.8 ± 15.8; 52.3 [42.0; 64.8]	0.002	0.423	−2.53 [−7.16; 2.10]	0.277
**Control and powerlessness**	60.1 ± 19.9; 58.3 [47.9; 75.0]	58.3 ± 20.6; 58.3 [44.5; 77.1]	0.408	58.5 ± 17.4; 58.3 [47.9; 72.9]	54.6 ± 16.8; 54.2 [45.8; 64.6]	0.099	0.410	−2.42 [−8.49; 3.64]	0.425
**Emotional well-being**	59.6 ± 21.4; 58.3 [43.8; 77.1]	54.2 ± 19.1; 50.0 [41.7; 68.8]	0.033	54.8 ± 19.4; 54.2 [39.6; 70.8]	49.0 ± 18.1; 45.8 [35.4; 64.6]	0.113	0.885	−1.86 [−9.29; 5.57]	0.616
**Social support**	57.5 ± 27.1; 68.8 [31.3; 81.3]	55.8 ± 22.6; 62.5 [31.3; 75.00]	0.604	42.2 ± 29.2; 31.3 [18.8; 71.9]	40.5 ± 25.8; 31.3 [18.8; 62.5]	0.170	0.448	−3.51 [−10.40; 3.39]	0.311
**Self-image**	55.9 ± 24.7; 58.3 [37.5; 75.0]	51.0 ± 23.4; 50.0 [33.3; 66.7]	0.053	51.1 ± 21.8; 50.0 [37.5; 66.7]	48.9 ± 21.9; 50.0 [37.5; 63.6]	0.285	0.519	3.74 [−3.78; 11.26]	0.322

Data are presented as mean ± SD and median [IQR]; SD, standard deviation; IQR, interquartile range; T0, baseline; T2, end of intervention; CG, control group; IG, intervention group; EHP-30, Endometriosis Health Profile-30. Within-group comparisons were performed using paired *t*-test or Wilcoxon signed-rank test; between-group Δ(T2 − T0) comparisons were performed using independent *t*-test or Mann–Whitney U test; ANCOVA: T2~group + T0; effect reported as (IG − CG) with 95% CI; CI, confidence interval; Δ, change from baseline to week 12; *p*-value < 0.05.

## Data Availability

The data that support the findings of this study are available on request from the corresponding author. The data are not publicly available due to privacy or ethical restrictions.

## Supplementary Materials

The following supporting information can be downloaded at: https://www.mdpi.com/article/10.3390/nu18132184/s1. Table S1: Detailed composition of the fish-derived lipid supplement (FDL) used in the study. Table S2: The categorical adequacy analysis of dietary fat intake.
